# In Vitro Root Induction from Argan (*Argania spinosa* (L.) Skeels) Adventitious Shoots: Influence of Ammonium Nitrate, Auxins, Silver Nitrate and Putrescine, and Evaluation of Plantlet Acclimatization

**DOI:** 10.3390/plants10061062

**Published:** 2021-05-26

**Authors:** Ilham Amghar, Mohammed Ibriz, Maha Ibrahimi, Abdelaali Boudra, Fatima Gaboun, Reda Meziani, Driss Iraqi, Mouaad Amine Mazri, Ghizlane Diria, Rabha Abdelwahd

**Affiliations:** 1 UR Biotechnologie, CRRA-Rabat, Institut National de la Recherche Agronomique, BP 6570, Rabat 10101, Morocco; amghar.ilham@gmail.com (I.A.); maha.ibrahimi@gmail.com (M.I.); gabounf@gmail.com (F.G.); iraqid@yahoo.fr (D.I.); ghizlanediria@gmail.com (G.D.); 2 Laboratoire de Génétique et Biométrie, Département de Biologie, Faculté des Sciences de Kénitra, Université Ibn Tofail, BP 133, Kenitra 14000, Morocco; m_ibriz@yahoo.fr; 3 Office National du Conseil Agricole, Agadir 80000, Morocco; boudra_1999@yahoo.fr; 4 Laboratoire National de Culture des Tissus du Palmier Dattier, UR Systèmes Oasiens, CRRA-Errachidia, Institut National de la Recherche Agronomique, Avenue Moulay Ali Cherif, BP 2, Errachidia 52000, Morocco; redameziani@yahoo.fr; 5 Laboratoire de Biotechnologie Végétale, UR Agro-Biotechnologie, CRRA-Marrakech, Institut National de la Recherche Agronomique, BP 533, Marrakech 40000, Morocco; m.a.mazri@gmail.com

**Keywords:** micropropagation, organogenesis, plant regeneration, rhizogenesis

## Abstract

*Argania spinosa* (L.) Skeels is an endangered plant species endemic to Morocco. In recent years, attempts to develop in vitro regeneration systems for this species were made. However, rooting and acclimatization of in vitro plants have been a bottleneck for successful propagation. In the present study, the effects of different concentrations of auxins, putrescine, silver nitrate (AgNO_3_) and ammonium nitrate on the in vitro rooting of adventitious shoots of two argan genotypes “Mejji” and “R’zwa”, were evaluated. The highest rooting percentages (86.6% in “Mejji” and 84.4% in “R’zwa”) were observed on Murashige and Skoog (MS) medium modified by reducing the ammonium nitrate concentration and supplemented with 1.5 mg L^−1^ indole-3-butyric acid (IBA), 0.5 mg L^−1^ 1-naphthalene acetic acid (NAA), 2 mg L^−1^ AgNO_3_ and 160 mg L^−1^ putrescine. This medium resulted in the development of a good root system after only 10 days of culture. Plantlet acclimatization was carried out using different substrate mixtures, and high survival rates (100%) were observed when the substrate contained either peat alone or a sand–peat mixture (1:1, *w*/*w*). The high percentages of rooting and acclimatization reported in the present study are of high importance for rapid and large-scale propagation of this endangered species.

## 1. Introduction

Argan (*Argania spinosa* (L.) Skeels) is a forest species belonging to the family Sapotaceae growing endemically in Morocco [1,2]. Argan plays multiple socioeconomic and environmental roles. In fact, argan fruits are used by local populations for the production of argan oil, a product that has long been valued for its nutritional, medicinal and therapeutic properties [3,4]. Argan oil is one of the most expensive and sought-after oils in the world. The price of argan oil in the international market exceeds US$400 per liter [5]. The argan oil industry significantly contributes to the income of local populations and sustains and improves their livelihood [6]. On the other hand, the argan ecosystem was reported to improve water quality, crop production and rangeland conditions and contributes to maintaining high soil quality, protecting biodiversity and controlling desertification [7].

Despite the high socioeconomic and ecological importance of argan, this species is threatened by several biotic and abiotic factors that led to the argan ecosystem’s degradation. For example, argan is overexploited by local people for food, wood, cosmetic and medicine industries [8]. In addition, argan suffers from intensive overgrazing by goats and natural habitat loss by urban expansion [8,9,10]. Developing efficient propagation systems for argan is today a key tool in the preservation and rehabilitation of the argan ecosystem.

The natural propagation of argan by seed germination and seedling growth is constrained by a wide range of factors. The most important among them is that argan is a slow-growing species; thus, seedlings hardly survive harsh environmental conditions and are permanently exposed to the risk of goat overgrazing [9,11]. Vegetative propagation through stem cuttings cannot be envisaged for the preservation and large-scale multiplication because of the recalcitrance of argan plants to rooting [12,13]. In vivo grafting is another approach used for the propagation of superior argan genotypes. The success of this technique depends on the environmental conditions, the degree of compatibility between the rootstock and scion and the physiological activity of scions [14].

Plant cell and tissue culture is a powerful tool for rapid and large-scale propagation of endangered plant species [15,16]. In the case of argan, this technology has not been well explored even though some studies were recently published [13,17,18,19]. Koufan et al. [17] and Lamaoui et al. [19] evaluated the effects of different plant growth regulators (PGRs) on bud break, shoot multiplication, elongation and adventitious root induction from argan microcuttings and concluded that the application of this technique is hindered by the difficulty to induce root formation from microcuttings. Koufan et al. [13] have described a novel approach for argan micropropagation by using in vitro grafting. This technique allows overcoming the rooting problems of microcuttings. However, plantlet survival during acclimatization should be improved [13]. Developing efficient regeneration systems through somatic embryogenesis or organogenesis will undoubtedly contribute to the propagation, genetic improvement and preservation of argan, as was the case in other oil crops [20,21,22,23]. Recently, a regeneration system has been established for argan through organogenesis [24]. The development of an efficient and reproducible organogenesis system strongly depends on successful in vitro rooting of the regenerated shoots and high survival rates during acclimatization to ex vitro conditions.

Rooting is a crucial step in the micropropagation of plant species [25]. The root system plays important roles in water and nutrient uptakes, gas transport processes, plant growth and development and in the defense mechanisms against biotic and abiotic stresses [26,27]. The in vitro rooting process is governed by diverse genetic factors and is greatly influenced by culture medium components, mainly the type and concentration of PGRs and polyamines [28,29,30]. On the other hand, plantlet survival in ex vitro conditions is significantly affected by the planting substrate, which is one of the main factors determining plant growth and development [31,32]. In argan, different potting substrates were used for the acclimatization of micropropagated plants. Nouaim et al. [12] planted argan microcuttings in a substrate composed of Terragreen saturated with “Long Ashton” nutrient solution. Koufan et al. [13] and Lamaoui et al. [19] used a mixture of peat and sand, while Justamante et al. [18] used a peat–perlite mix.

The present study aimed to determine the effects of nitrate, putrescine, and PGRs on in vitro root induction and growth from adventitious shoots of two argan genotypes, “Mejji” and “R’zwa”, and to select the best planting substrate for improved plant survival under ex vitro conditions.

## 2. Results

### 2.1. Effects of Putrescine, Silver Nitrate (AgNO_3_), Ammonium Nitrate (NH_4_NO_3_), Indole-3-Butyric Acid (IBA) and 1-Naphthalene Acetic Acid (NAA) on In Vitro Rooting of Regenerated Shoots

#### 2.1.1. Effects of NH_4_NO_3_ on In Vitro Rooting

The concentration of NH_4_NO_3_ had a significant effect on argan shoot rooting (Table 1). Indeed, when the culture media were supplemented with both IBA and putrescine, Murashige and Skoog (MS) medium showed significantly higher rooting percentages (52.2% in “Mejji” and 57.7% in “R’zwa”) than RM (MS modified by reducing NH_4_NO_3_ concentration to 825 mg L^−1^) and M (MS without NH_4_NO_3_) media. However, the shoots cultured on RM medium showed the highest average number of roots after 75 days of culture (4.6 in “Mejji” and 4.4 in “R’zwa”). In the M medium, significantly lower rooting percentages were observed (Table 1).

#### 2.1.2. Effects of IBA, AgNO_3_ and Putrescine on In Vitro Rooting

**Figure 1:**
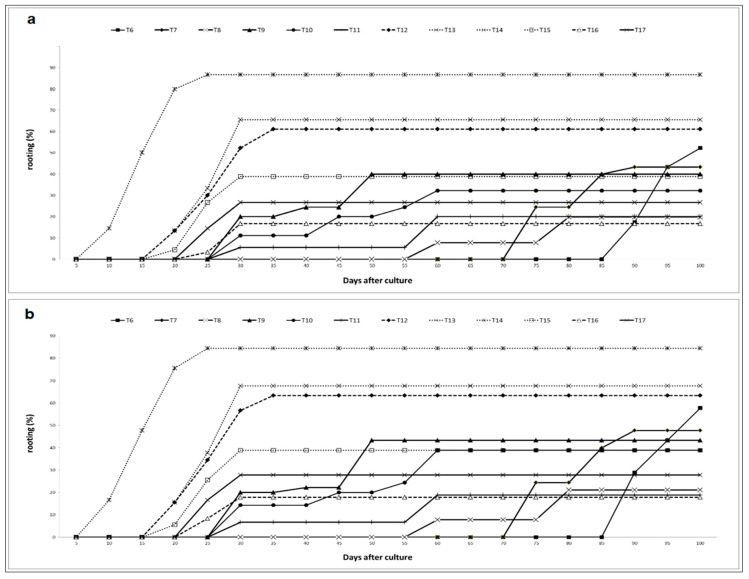
In vitro rooting kinetic of the two genotypes of *Argania Spinosa* in different culture media mentioned in Table 1. (**a**) genotype “Mejji”; (**b**) genotype “R’zwa”.

**Figure 2:**
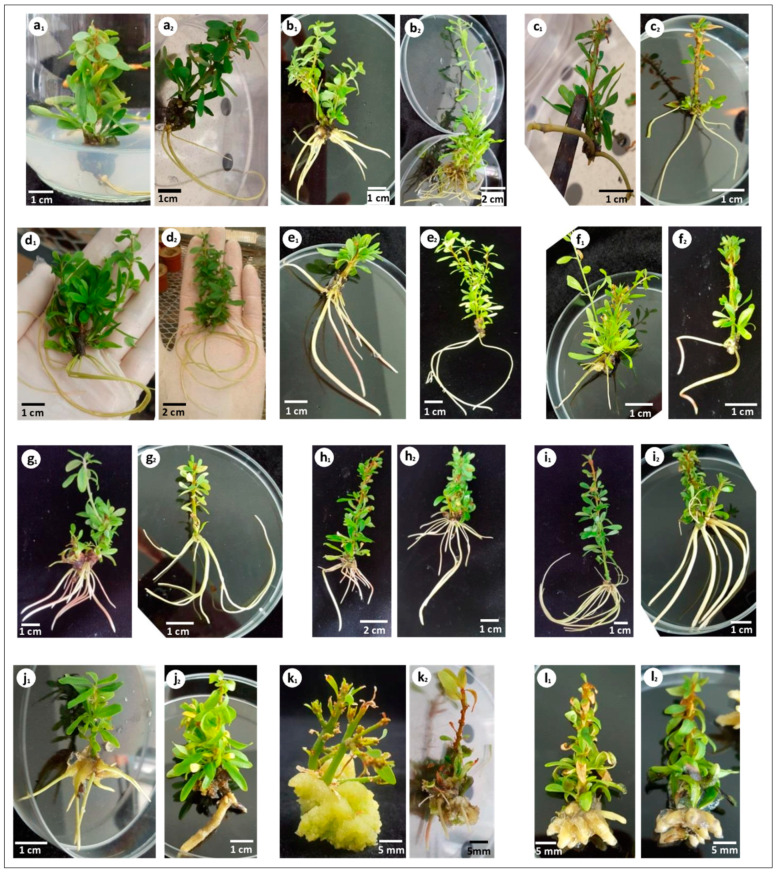
In vitro rooting of different argan genotypes. (**a**) In vitro rooting in the presence of putrescine and indole-3-butyric acid (IBA) in Murashige and Skoog (MS) medium; (**b**) in vitro rooting in the presence of putrescine and IBA in MS medium modified by reducing ammonium nitrate concentration to 825 mg L^−1^ (RM medium); (**c**) in vitro rooting in the presence of putrescine and IBA in MS medium without ammonium nitrate (M medium); (**d**) in vitro rooting in the presence of silver nitrate (AgNO_3_) (2 mg L^−1^) and IBA; (**e**) in vitro rooting in the presence of AgNO_3_ (4 mg L^−1^) and IBA; (**f**) in vitro rooting in the presence of AgNO_3_ (6 mg L^−1^) and IBA; (**g**) in vitro rooting in the presence of AgNO_3_ (2 mg L^−1^), putrescine and IBA; (**h**) in vitro rooting in RM medium containing AgNO_3_ (2 mg L^−1^), putrescine and IBA; (**i**) in vitro rooting in RM medium containing AgNO_3_ (2 mg L^−1^), putrescine, IBA and 1-naphthalene acetic acid (NAA) (0.5 mg L^−1^); (**j**) in vitro rooting in RM medium containing AgNO_3_ (2 mg L^−1^), putrescine, IBA and NAA (1 mg L^−1^); (**k**) in vitro rooting in RM medium containing AgNO_3_ (2 mg L^−1^), putrescine, IBA and NAA (1.5 mg L^−1^); (**l**) in vitro rooting in RM medium containing AgNO_3_ (2 mg L^−1^), putrescine and NAA (1.5 mg L^−1^). (**_1_**) indicates genotype “Mejji”, and (**_2_**) indicates genotype “R’zwa”.

In both argan genotypes, no root formation was observed in auxin-free media. Likewise, the use of either putrescine, AgNO_3_ or IBA alone did not induce adventitious rooting in micropropagated shoots. Rooting occurred in shoots cultured on media containing at least one auxin (IBA or NAA) plus putrescine and/or AgNO_3_ (Table 1). The shoots cultured on MS medium supplemented with 1.5 mg L^−1^ IBA and 160 mg L^−1^ putrescine showed rooting percentages of 57.7% and 52.2% in “R’zwa” and “Mejji”, respectively. In all putrescine-IBA-containing media, root induction required 8 weeks, while 2 more weeks were needed for root development (Figure 1). In some cases, callus formation was observed at the cut end of shoots. This results in low-quality roots, not suitable for acclimatization (Table 1 and Figure 2).

In MS medium, the combination of AgNO_3_ and IBA resulted in rooting percentages ranging from 19.9 to 39.9% in genotype “Mejji”, and from 18.8 to 43.3% in genotype “R’zwa”, depending on AgNO_3_ concentration. In fact, AgNO_3_ concentration significantly affected root induction, as well as the average number of roots per shoot and root length (Table 1). Increasing the concentration of AgNO_3_ to 4 and 6 mg L^−1^ decreased the rooting percentage and the average number of roots per shoot (Table 1).

#### 2.1.3. Evaluation of the Combined Effects of Putrescine, AgNO_3_, IBA and NAA on In Vitro Rooting

To improve in vitro rooting and the quality of the root system, the combined effect of NH_4_NO_3_, putrescine, AgNO_3_, IBA and NAA was evaluated (Figure 2). The results showed that combining IBA, putrescine and 2 mg L^−1^ AgNO_3_ significantly reduced the root induction time to 20 days in MS and RM media in both argan genotypes. Adding NAA at a low concentration (0.5 mg L^−1^) resulted in the highest rooting percentages (86.6% in “Mejji” and 84.4% in “R’zwa”), an average root length of 3.5 cm in “Mejji” and 3.4 cm in “R’zwa”, and an average number of roots per shoot of 6.3 in “Mejji” and 6.7 in “R’zwa”. This combination showed root emergence within the first 10 days of culture (Figure 1). The average number of roots per shoot depended on NAA concentration, with the highest number of roots per shoot (10.1 in “Mejji” and 10.5 in “R’zwa”) observed when 1.5 mg L^−1^ NAA was added (Table 1). However, the use of 1. 5 mg L^−1^ NAA resulted in high callogenesis and low root quality.

#### 2.1.4. Effects of Putrescine, AgNO_3_, IBA and NAA on Rooting Kinetics

The results of the present study showed that culture medium components have a significant impact on root induction. Shoot rooting started after 55 to 85 days of culture in media supplemented with putrescine, whereas, in media supplemented with AgNO_3_, rooting started after 25 days of culture. In both genotypes, when the shoots were cultured on RM medium supplemented with 160 mg L^−1^ putrescine, 2 mg L^−1^ AgNO_3_, 1.5 mg L^−1^ IBA and 0.5 mg L^−1^ NAA, rooting was observed after only 5 days of culture (Figure 1). Moreover, the rooting percentage reached 50% and 80% in genotype “Mejji” after 15 and 20 days of culture, respectively.

### 2.2. Effect of Different Substrate Mixtures on Ex Vitro Acclimatization

The substrate significantly influenced the survival rate of the regenerated plantlets (Table 2). The highest survival rate (100%) was observed when the plantlets of both genotypes were transplanted into peat substrate or in the peat–sand mixture. When the plantlets were transplanted into a sand substrate, the survival rate differed between the two genotypes (Table 2). In fact, a survival rate of 100% was observed in “Mejji” plantlets, while the plantlets of “R’zwa” showed a survival rate of 86.6%. The substrate mixture composed of peat, sand, and forest soil showed lower survival rates (73.3 and 83.3% in “Mejji” and “R’zwa”, respectively).

**Figure 3:**
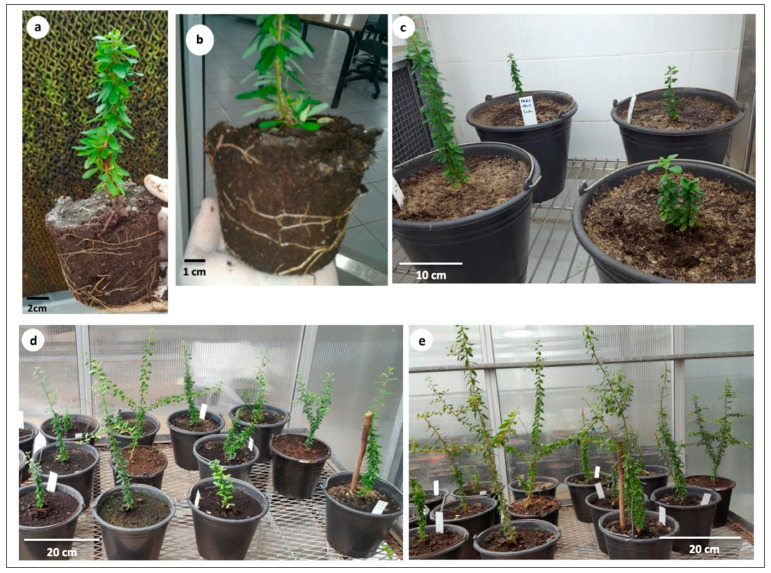
Acclimatization of argan plantlets. (**a**–**c**) plantlets of genotypes “Mejji” (**a**) and “R’zwa” (**b**,**c**) after 4 weeks in the glasshouse. (**d**) Plantlet growth after 10 weeks in the glasshouse. (**e**) Plantlet growth after 16 weeks in the glasshouse.

The substrate also influenced the plant growth during acclimatization (Table 2 and Figure 3). In sand substrate, there was a significant difference between the two genotypes, “Mejji” and “R’zwa”. In fact, the average stem growth per plantlet was 2.7 cm in “Mejji” and 0.8 cm in “R’zwa” (Table 2). The highest stem growth (2.0 cm) in genotype “R’zwa” was observed in peat substrate.

The effect of the substrate mixture differed between the two genotypes. The use of peat–sand mixture or sand alone showed the formation of an average of 0.4 shoots per plantlet in “Mejji”, while no new shoots were observed in “R’zwa” (Table 2). The use of peat alone did not promote the formation of new shoots in both genotypes, whereas the peat, sand, and forest soil mixture was the only substrate that promoted new shoot formation in both genotypes (Table 2). The plantlets grown in peat alone exhibited vigorous growth, green leaves, and well-developed and elongated secondary roots.

## 3. Discussion

Argan tree is one of the most recalcitrant species in tissue culture. Indeed, the adventitious root formation is very difficult to achieve, while it is critical for successful vegetative propagation of this species [12]. The development of healthy and vigorous roots is essential for successful acclimatization to ex vitro conditions. In a previous study by our group [24], adventitious shoot rooting was achieved on media containing both IBA (1.5 mg L^−1^) and putrescine (160 mg L^−1^). The beneficial effects of polyamines in general and putrescine in particular on rooting were reported in many other plant species, such as olive [30,33], hazelnut [34], almond [35] and teak [36]. Cristofori et al. [37] showed that putrescine, spermidine and spermine improve root formation of microshoots treated with IBA. Many other authors (e.g., Chilley et al. [38]; Weiss and Ori [39]; Saini et al. [40]) reported that products, such as jasmonic acid, strigolactones and polyamines interact either synergistically or antagonistically with auxins to trigger different types of events that lead to root formation and development. On the other hand, Cristofori et al. [37] reported that polyamines have a limited positive effect on rooting when applied without IBA. This is in good agreement with our findings. In fact, the results of the present study showed that using putrescine alone did not promote root induction in argan shoots. Moreover, it was found that putrescine induces callus formation, which is probably due to its effect on cell division [41,42]. According to Hartmann and Kester [43] and Hartmann et al. [44], root formation can be seen from established calli or not. In the present study, callus formation was higher in shoots cultured on RM medium supplemented with putrescine, IBA and 1.5 mg L^−1^ NAA. Moreover, the roots initiated from calli were fragile and easily detachable.

Our results also showed that combining IBA and NAA resulted in a higher rooting frequency than that observed when IBA was used alone. However, increasing NAA concentration decreased the rooting percentage. This highlights the specific requirement of argan shoots in terms of auxin types and concentrations for efficient in vitro rooting. The effects of NAA and IBA on root induction have been evaluated by different researchers during both in vitro and conventional propagation of argan. Justamante et al. [18] reported that the combination of 1 mg L^−1^ NAA and 1 mg L^−1^ IBA inhibited adventitious rooting in seedling-derived shoots. However, in shoots produced vegetatively from microcuttings, Lamaoui et al. [19] recommended the combination of 5 mg L^−1^ IBA and 1 mg L^−1^ NAA for root induction, whereas Koufan et al. [17] recommended the combination of 0.5 mg L^−1^ NAA and 0.5 mg L^−1^ 6-benzylaminopurine (BAP). On the other hand, Benbya et al. [45] found that IBA is more effective than NAA in inducing adventitious roots from semi-hard wood cuttings. These divergent results could reflect different hormonal requirements among argan genotypes and explant types (juvenile, rejuvenated and adult material).

The effects of AgNO_3_ on in vitro rhizogenesis were also investigated. The findings of the present study showed that AgNO_3_ promotes root formation and the development of vigorous roots within 30 days of culture, with no callus formation. The optimal concentration of AgNO_3_ was 2 mg L^−1^. This concentration improved the root system of shoots, which results in increased plantlet survival and better growth and development. In many plant species, such as *Prosopis cineraria* [46], *Vanilla planifolia* [47], *Coffea arabica*, *C. canephora* [48], and *Rotula aquatica* [49], AgNO_3_ has improved in vitro rooting. Silver nitrate is known to be an ethylene inhibitor. In fact, Ag+ ions produced in the culture medium play the role of an ethylene receptor, thus ensuring easy assimilation of the energy available in the culture medium, which improves cell division and growth. Venkatachalam et al. [46] reported a similar effect of AgNO_3_ on rooting of the recalcitrant medicinal tree *Prosopis cineraria*. Klíma et al. [50] showed that silver ions increase plasma membrane permeability for water and small organic compounds as well as auxin efflux. Kumar et al. [51] reported in their review that adding silver ions to culture medium regulates the ethylene activity and improves organogenesis, in vitro rooting, flowering and shoot development. Nonetheless, the mechanism of action of silver ions remains unclear. According to Kumar et al. [51], silver ions target functionally interlinked ethylene, polyamine and calcium-mediated pathways. Ruzicka et al. [52] indicated that ethylene supports auxin biosynthesis and modulates its distribution in *Arabidopsis* seedling roots. They also demonstrated that silver ions could block ethylene-induction in auxin changes. Bais [53] reported that AgNO_3_ exerts a feedback inhibition on ethylene synthesis and stimulates polyamine biosynthesis by an enhanced use of S-adenosylmethionine. Ethylene can also influence callus formation [54]. These combined actions are probably due to an interplay between polyamine and ethylene biosynthesis, which regulates in vitro morphogenetic responses [55].

The findings of the present investigation showed that successful in vitro rooting and development of vigorous roots in argan shoots was obtained when NH_4_NO_3_ concentration was reduced to 825 mg L^−1,^ and the culture medium was supplemented with auxins (IBA and NAA), putrescine and AgNO_3_. This results in vigorous root development within only 10 days of culture. This reflects the specific requirements of argan microshoots for root induction. In fact, some components of the culture medium allow more cells to be used for root induction, which improves the rooting ability of shoots [56]. Similar results were observed in other species. The beneficial effect of low NH_4_NO_3_ concentration on in vitro rooting was first demonstrated in apple [57]. Vahdati et al. [58] found that media with doubled NH_4_NO_3_ concentration reduced the rooting ability of Persian walnut. In *Lavandula* spp., rooting was promoted by reducing the concentration of MS micronutrients to ½ or ¼ and by adding NAA to the culture medium [59]. Similarly, the use of auxin-containing MS medium with macroelements reduced to half, to one-third or to one-quarter strength was effective for root induction in *Rosa* spp [60].

Plantlet acclimatization is a crucial step that determines the efficiency of the whole micropropagation process. During acclimatization, regenerants are exposed to new growth conditions causing biotic and abiotic stresses, such as water loss, tissue dehydration and synthesis process reduction [61]. According to many authors (e.g., Lone et al. [31]; Stefanello et al. [32]), the substrate used during acclimatization has a significant effect on the survival and growth of the plantlets regenerated through in vitro culture. The potting substrate is an essential factor in the success of plantlet acclimatization. In previous studies on the micropropagation of argan, different substrates were used. Koufan et al. [13] planted argan micrografted plants in a mixture of peat and sand. Regarding argan microcuttings, Nouaim et al. [12] used a substrate composed of Terragreen saturated with “Long Ashton” nutrient solution. Lamaoui et al. [19] used a mixture of peat and sand, while Justamante et al. [18] used a peat–perlite mix. The findings of the present study showed that the argan plantlets grown in peat–sand mixture or in peat alone exhibit remarkable adaptation to ex vitro conditions as well as good growth and development. The significant impact of the substrate on plant survival and growth can be explained by its physicochemical properties. In fact, the substrate must be porous and well-drained. In addition, it should facilitate the acquisition of water and nutrients and allow gas exchange. This will promote plant growth and survival and the development of a good root system [62].

## 4. Materials and Methods

### 4.1. Culture Conditions

The basal medium used in the present study was that of MS medium [63] containing MS macroelements, MS microelements and MS vitamins. Moreover, modified MS media were also used: RM, Murashige and Skoog basal medium modified by reducing NH_4_NO_3_ concentration to 825 mg L^−1^; and M, Murashige and Skoog basal medium modified by omitting NH_4_NO_3_. All media were supplemented with 30 g L^−1^ sucrose and 8 g L^−1^ agar. The medium pH was adjusted to 5.8 before autoclaving at 120 °C and 103 kPa for 20 min. The chemicals used in this study were purchased from Sigma (St. Louis, MO, USA).

### 4.2. Plant Material and Experiments

#### 4.2.1. Origin of Plant Material

Mature seeds of argan genotypes “Mejji” and “R’zwa” were harvested from trees located in Essaouira province, Morocco. The seeds of the genotype “R’zwa” were collected from a tree located approximately 7 km southeast of Essaouira city (31°26′48.17″ N 9°43′43.74″ W), while those of the genotype “Mejji” were collected from a tree located about 45 km east of Essaouira city (31°32′49.71″ N 9°22′32.44″ W). From each tree, 100 seeds were collected.

**Figure 4:**
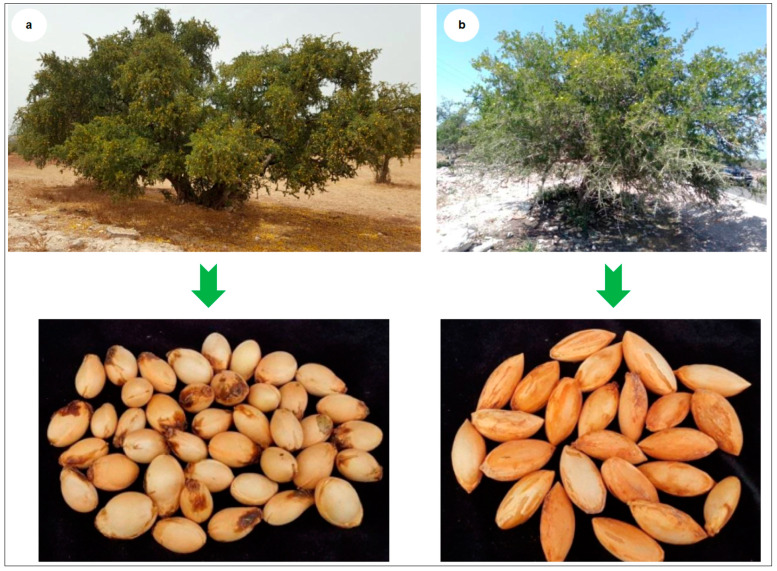
Argan trees and seeds. (**a**) genotype “Mejji” and (**b**) genotype “R’zwa”.

Both the trees are characterized by high fruit yield. However, they are morphologically different. Genotype “Mejji” has a bigger size and produces round-shaped fruits, while genotype “R’zwa” produces ellipsoid-shaped fruits (Figure 4).

The seeds were surface-sterilized by immersion in 70% ethanol for 2 min, followed by immersion in 30% sodium hypochlorite solution containing a few drops of Tween-20 for 15 min. The seeds were then immersed in 0.1% mercury chloride for 10 min, followed by 3 rinses with sterile distilled water, then by immersion in 0.2% polyvinylpyrrolidone solution for 60 min [24]. To promote germination, the seeds were soaked in 10 mg L^−1^ GA_3_ for 12 h and then cultured for 35 days on sucrose-free 1/2MS medium modified by omitting ammonium nitrate [24].

**Figure 5:**
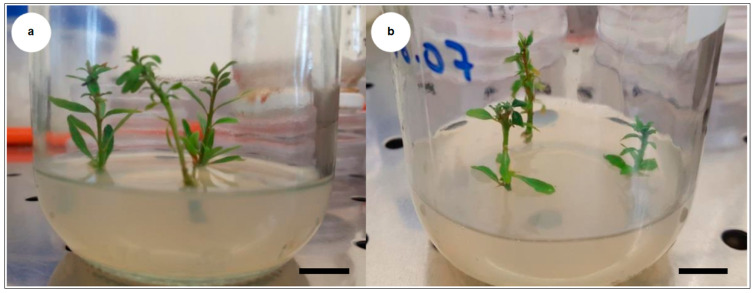
Adventitious shoots of argan used for rhizogenesis experiments. (**a**) genotype “R’zwa” and (**b**) genotype “Mejji”. Bars correspond to 1 cm.

The epicotyl explants were excised from in vitro germinated seedlings and cultured on semi-solid MS medium supplemented with 2 mg L^−1^ BAP for 4 weeks under dark conditions to induce organogenesis. The organogenesis rate was 79.1% (1.8 shoot buds per explant) in “Mejji” and 62.5% (1.9 shoot buds per explant) in “R’zwa” [24]. For shoot bud proliferation, the organogenic cultures were transferred to MS medium supplemented with 1 mg L^−1^ BAP and 2 mg L^−1^ gibberellic acid (GA_3_) for 4 weeks under a 16 h photoperiod [24]. The adventitious shoots obtained through this organogenesis pathway were used in the present study (Figure 5).

#### 4.2.2. In Vitro Rhizogenesis

Healthy shoots (2–3 cm long) of argan genotypes “Mejji” and “R’zwa”, obtained through direct organogenesis as previously described, were singled out and cultured on PGR-free MS medium for 2 weeks. Subsequently, the shoots were transferred to different rooting media (MS or modified MS media) supplemented with various concentrations of IBA (0 or 1.5 mg L^−1^), NAA (0, 0.5, 1 or 1.5 mg L^−1^), putrescine (160 mg L^−1^) and/or AgNO_3_ (2, 4 or 6 mg L^−1^) (Table 1). The explants were placed in 300 mL glass jars (12 cm in height, 6.5 cm in diameter) containing 40 mL of culture medium and sealed with transparent plastic lids. The cultures were kept at 25 °C ± 1 in darkness for 7 days and then transferred to a 16 h photoperiod (40 μmol m^−2^ s^−1^ provided by cool white fluorescent tubes). This experiment lasted for 100 days, and the cultures were transferred to fresh medium at 4-week intervals. The percentage of shoots forming roots, the mean number of roots per shoot and the average length of roots were recorded every 5 days of culture. Moreover, the amount of callus formed was visually estimated as follows: 0C: no callus, C+: low amount of callus, C++: moderate callogenesis, C+++: high amount of callus. The time required for root induction was determined by visually inspecting the cultures every 5 days. The experiment was performed in three replicates, with 30 shoots per treatment, and was repeated twice.

#### 4.2.3. Ex Vitro Acclimatization

The rooted plantlets obtained from the best treatment were removed from the rooting medium, and their roots were carefully washed with distilled water to remove adhered agar. The plantlets were then transferred to plastic pots (one plant per pot) containing autoclaved mixtures of sand, peat and forest soil in different proportions 1:0:0, 0:1:0, 1:1:1, and 1:1:0 (*w*/*w*/*w*) and placed in the growth room. To maintain high humidity, the pots were covered with transparent polyethylene bags. After 2 weeks, the plantlets were transferred to the glasshouse. They were maintained at 25 ± 2 °C under light conditions and regularly irrigated with Hoagland and Arnon’s nutrient solution [64]. Meanwhile, the polyethylene bags were gradually removed. Finally, the plantlets were transferred to larger plastic pots. After 4 weeks in the glasshouse, the survival rate, the number of neoformed shoots, and the plant growth (i.e., the initial size of the plantlets and the size after 28 days of transplantation) were recorded. Three replicates of 5 pots each were used per substrate, and the experiment was repeated twice.

### 4.3. Data Analysis

Data are expressed as means and standard deviations (SD). All experiments were set up in a completely randomized block design. One-way analysis of variance (ANOVA I) was performed to test for significant differences among the different treatments. The least significant difference test (LSD) was used for post hoc mean separation at the 5% significance level. Tests for normality and homogeneity of variance were performed before ANOVA. Before analysis, all percentage data were arcsine transformed.

## 5. Conclusions

We described an efficient protocol for in vitro rooting and acclimatization of micropropagated argan shoots. The optimal medium for shoot rooting was RM medium (MS medium modified by reducing NH_4_NO_3_ concentration to 825 mg L^−1^) supplemented with 160 mg L^−1^ putrescine, 2 mg L^−1^ AgNO_3_, 1.5 mg L^−1^ IBA and 0.5 mg L^−1^ NAA. In this medium, rooting started after only five days of culture. Moreover, the development of a healthy and vigorous root system was observed, with no or low callus formation. In fact, adding putrescine, NAA, IBA and AgNO_3_ promoted root formation, inhibited callogenesis and reduced the time required to produced rooted plantlets ready for acclimatization. AgNO_3_ had an inhibitory effect on callus formation and improved the quality of the root system. The addition of NAA at low concentration increased the percentage of rooting and the number and length of roots. Regarding acclimatization, a high survival rate of 100% and normal growth and development of plants were observed in the peat–sand mixture or when peat was used alone. The reported results will be used for rapid and large-scale propagation of this endangered plant species and to rehabilitate the argan ecosystem. Further studies are currently undertaken to test the developed protocol’s suitability on other superior argan genotypes characterized by high fruit yield and high oil content.

## Figures and Tables

**Table 1:** Effect of genotype, putrescine, silver nitrate, indole-3-butyric acid (IBA) and 1-naphthalene acetic acid (NAA) on rooting percentage, number of roots per shoot, root length, callus formation and the time required for rooting in two argan (*Argania spinosa* (L.) Skeels) genotypes, “Mejji” and “R’zwa”.

Treatment	Basal Medium	AgNO_3_ (mg L^−1^)	Putrescine (mg L^−1^)	PGRs (mg L^−1^)		“Mejji”					“R’zwa”				
				IBA	NAA	Rooting Frequency (%)	Average No. of Roots/Shoot	Average Root Length (cm)	Callus Formation	Time for Rooting (Days)	Rooting Frequency (%)	Average No. of Roots/Shoot	Average Root Length (cm)	Callus Formation	Time for Rooting (Days)
T0	MS	-	-	-	-	0	0	0	0C	-	0	0	0	0C	-
T1	MS	-	-	1.5	-	0	0	0	0C	-	0	0	0	0C	-
T2	MS	-	160	-	-	0	0	0	0C	-	0	0	0	0C	-
T3-T4-T5	MS	2002/4/6	-	-	-	0	0	0	0C	-	0	0	0	0C	-
T6	MS	-	160	1.5	-	52.2 ± 1.1 ^c^	3.1 ± 0.2 ^d,e^	2.8 ± 0.2 ^a^	C+++	90	57.7 ± 2.9 ^c^	2.8 ± 0.2 ^a^	3.4 ± 0.2 ^a,b,c^	C+++	90
T7	RM	-	160	1.5	-	43.3 ± 3.8 ^d^	4.6 ± 0.2 ^c^	3.1 ± 0.2 ^a^	C++	75	47.7 ± 2.9 ^d^	4.4 ± 0.2 ^c^	2.8 ± 0.2 ^a,b,c^	C+++	75
T8	M	-	160	1.5	-	19.9 ± 1.9 ^f,g^	2.5 ± 0.3 ^e,f,g^	2.0 ± 0.1 ^b,c^	C++	60	21.1 ± 2.2 ^f,g^	2.8 ± 0.3 ^e,f^	3.0 ± 0.1 ^a,b,c^	C+++	60
T9	MS	2	-	1.5	-	39.9 ± 1.9 ^d^	3.3 ± 0.2 ^d,e^	3.3 ± 0.3 ^a^	0C	30	43.3 ± 1.9 ^de^	3.1 ± 0.2 ^d,e,f^	2.7 ± 0.2 ^a,b,c^	0C	30
T10	MS	4	-	1.5	-	32.2 ± 1.1 ^d,e^	1.9 ± 0.1 ^f,g^	1.6 ± 0.1 ^c^	0C	30	38.8 ± 1.1 ^e^	2.2 ± 0.1 ^f,g^	2.3 ± 0.1 ^c^	0C	30
T11	MS	6	-	1.5	-	19.9 ± 1.9 ^f,g^	1.9 ± 0.2 ^f,g^	2.8 ± 0.4 ^a,b^	0C	30	18.8 ± 2.9 ^g^	1.5 ± 0.1 ^g^	2.4 ± 0.3 ^b,c^	0C	30
T12	MS	2	160	1.5	-	61.1 ± 2.9 ^b^	3.2 ± 0.1 ^d,e^	3.3 ± 0.2 ^a^	0C	20	63.3 ± 3.3 ^b,c^	3.3 ± 0.1 ^c,d^	3.2 ± 0.1 ^a,b^	0C	20
T13	RM	2	160	1.5	-	65.5 ± 4.8 ^b^	3.7 ± 0.2 ^c,d^	3.2 ± 0.2 ^a^	0C	20	67.7 ± 4.8 ^c^	3.8 ± 0.2 ^c,d^	3.3 ± 0.2 ^a^	0C	20
T14	RM	2	160	1.5	0.5	86.6 ± 3.8 ^a^	6.3 ± 0.3 ^b^	3.5 ± 0.1 ^a^	0C	10	84.4 ± 2.9 ^a^	6.7 ± 0.3 ^b^	3.4 ± 0.1 ^a^	C+	10
T15	RM	2	160	1.5	1	38.8 ± 1.1 ^c,d^	2.9 ± 0.1 ^d,e,f^	3.2 ± 0.2 ^a^	C+	20	38.8 ± 1.1 ^e^	3.3 ± 0.2 ^d,e^	2.9 ± 0.2 ^a,b,c^	C++	20
T16	RM	2	160	1.5	1.5	16.6 ± 1.9 ^g^	1.6 ± 0.1 ^g^	1.4 ± 0.2 ^c^	C+++	25–30	17.7 ± 2.9 ^g^	2.6 ± 0.3 ^s,f^	1.4 ± 0.1 ^d^	C+++	25–30
T17	RM	2	160	-	1.5	26.6 ± 1.9 ^e,f^	10.1 ± 0.6 ^a^	1.6 ± 0.1 ^c^	C+++	25	27.7 ± 1.1 ^f^	10.5 ± 0.4 ^a^	1.5 ± 0.1 ^d^	C+++	25

Data are means ± SD. Values in the same column and with different letter(s) are significantly different at *p* < 0.05. T, Treatment. MS, Murashige and Skoog basal medium; RM, Murashige and Skoog basal medium, but contains 825 mg L^−1^ NH_4_NO_3_; M: Murashige and Skoog basal medium without NH_4_NO_3_. Callus formation: 0C, no callus; C+, low callus formation; C++, moderate callus formation; C+++, high callus formation.

**Table 2:** Effects of different substrate mixtures on survival rate, the number of neoformed shoots and stem growth during ex vitro acclimatization of argan plantlets.

Substrate Mixture Peat:Sand:Forest Soil	Survival Rate (%)		Average Number of Neoformed Shoots		Stem Growth (cm/Plant)		Stem Growth (cm/Week)	
	“Mejji”	“R’zwa”	“Mejji”	“R’zwa”	“Mejji”	“R’zwa”	“Mejji”	“R’zwa”
S1 = 1:1:1	73.3 ^b^ ± 6.6	83.3 ^b^ ± 3.3	0.2 ^a,b^ ± 0.1	0.2 ^b^ ± 0.1	1.9 ^a,b^ ± 0.2	1.3 ^a,b^ ± 0.2	0.4 ^a,b^ ± 0.0	0.3 ^a^ ± 0.0
S2 = 1:1:0	100.0 ^a^ ± 0.0	100.0 ^a^ ± 0.0	0.4 ^a^ ± 0.1	0.0 ^a^ ± 0.0 ǂ	0.9 ^b^ ± 0.2	0.9 ^a^ ± 0.2	0.2 ^b^ ± 0.0	0.2 ^a^ ± 0.0
S3 = 1:0:0	100.0 ^a^ ± 0.0	100.0 ^a^ ± 0.0	0.0 ^b^ ± 0.0	0.0 ^a^ ± 0.0	1.3 ^b^ ± 0.2	2.0 ^b^ ± 0.3	0.3 ^b^ ± 0.0	0.5 ^a^ ± 0.0
S4 = 0:1:0	100.0 ^a^ ± 0.0	86.6 ^b^ ± 3.3 ǂ	0.4 ^a^ ± 0.1	0.0 ^a^ ± 0.0 ǂ	2.7 ^a^ ± 0.6	0.8 ^a^ ± 0.1 ǂ	0.7 ^a^ ± 0.1	0.2 ^a^ ± 0.0 ǂ

Data are means ± SD. Values in the same column and with different letter(s) are significantly different at *p* < 0.05. ǂ indicates significance between genotypes at *p* < 0.05. Substrate mixtures: S1, peat + sand + forest soil; S2, peat + sand; S3, peat; S4, sand.
